# New species of subgenus Tipula (Sivatipula) from China, with redescription of T. (S.) parvauricula and a key to all known species of the Oriental Region (Diptera, Tipulidae, *Tipula*)

**DOI:** 10.3897/zookeys.563.7176

**Published:** 2016-02-15

**Authors:** Guo-Xi Xue, Qiu-Lei Men

**Affiliations:** 1 School of Food and Bioengineering, Zhengzhou University of Light Industry, No. 5 Dongfeng Road, Zhengzhou, Henan 450002, P. R. China; 2 School of Life Sciences, the Province Key Laboratory of the Biodiversity Study and Ecology Conservation in Southwest Anhui, Anqing Normal University, Anqing, Anhui 246011, P.R. China

**Keywords:** China, crane flies, new species, semen pump, *Sivatipula*, *Tipula*, Tipulidae

## Abstract

Species of Tipula (Sivatipula) biprocessa **sp. n.** from Guangxi, China is described and illustrated as new in the subgenus Tipula (Sivatipula) Alexander, 1964. Tipula (Sivatipula) parvauricula Alexander, 1941 is redescribed and illustrated based on additional morphological characters. Semen pump of this subgenus is discussed. A key to all described species in this group is compiled.

## Introduction

Tipula (Sivatipula) Alexander, 1964 is a small subgenus with *Tipula mitocera* Alexander, 1927 from the eastern Himalayas, India as its type species. The other species in this subgenus include Tipula (Sivatipula) pullimargo Alexander, 1951 from Myanmar, Tipula (Sivatipula) alhena Alexander, 1953 from Thailand, Tipula (Sivatipula) filicornis Brunetti, 1918 and Tipula (Sivatipula) bhishma Alexander, 1964 from India, Tipula (Sivatipula) lackschewitziana Alexander, 1928, Tipula (Sivatipula) suensoniana Alexander, 1940 and Tipula (Sivatipula) parvauricula Alexander, 1941 from China (Oosterbroek 2015). All these species are restricted to the Oriental region. The Chinese fauna of Tipula (Sivatipula) is poorly represented with only three known species.

This subgenus is characterized by the following characters: male with antennae very long, slightly shorter, equal to or longer than body length, female with antennae relatively short, not beyond half length of body, flagellomere covered with six or seven long strong verticils; wing with squama naked, outer wing veins scattered with small, abundant macrotrichia, R_1+2_ entire, Rs longer than m-cu; ninth tergite and sternite fused, median region of sternite extensive, more or less protrudent, forming amembranous extension; inner and outer gonostylus irregularly varied in shape. The species of subgenus Tipula (Sivatipula) had been placed previously in subgenus Tipula (Acutipula) Alexander, 1924, but treated subsequently as a distinct group based on the combined structural characters of antennae, hypopygium, and wing (Alexander 1964).

A previously unknown taxon of Tipula (Sivatipula) was noticed while sorting crane flies specimens collected from Leigongshan Mountain, Guizhou Province, and Cenwanglaoshan Mountain, Guangxi Zhuang Autonomous Region, China. In the present paper, the new species is described and illustrated, and a key is provided for separating all known species. Tipula (Sivatipula) parvauricula is redescribed based on newly available morphological characters with detailed illustrations. The original description of this species is insufficient and the illustrations are too simple to reveal necessary characters. The character of semen pump of subgenus Tipula (Sivatipula) is described for the first time. The current study also demonstrated the new distribution pattern for the subgenus Tipula (Sivatipula) in both Guangxi Zhuang Autonomous Region and Guizhou Province. Future collecting and investigation would undoubtedly increase the species numbers and range extension of this group in China.

## Material and methods

The genital segments of the specimens were soaked in 10% NaOH overnight and observed or drawn in glycerine using a Leica MZ125 (Leica, Germany) stereomicroscope. The genital segments were then preserved in glycerine in 0.20 ml centrifuge tubes. Photographs of partial body of male were taken by Canon 5D Mark II digital single lens reflex camera (Canon, Japan) with MP-E 65mm f/2.8 1-5X macro lens (Canon, Japan). All measurements are in millimeters (mm), made with the aid of a digital caliper. The terminology and methods of description and illustration follow those of Alexander and Byers (1981) and Frommer (1963). The type specimens of the new species are deposited in the animal specimen room, School of Life Sciences, Anqing Normal University, Anqing, Anhui Province, China.

The key was principally constructed from descriptions in the literature without examination of the type species of most of these species, and should be considered preliminary. The characters used in the key rely primarily on the structures of genitalia and the length of antenna of male specimens.

## Taxonomy

### Tipula (Sivatipula) parvauricula

Taxon classificationAnimaliaDipteraTipulidae

Alexander, 1941

Figs 1–11

Tipula parvauricula Alexander, 1941: 400 (original description), Pl. 1, fig. 14, Pl. 4, fig. 44.

#### Diagnosis.

Generally orange-yellow in coloration; antenna distinctly longer than body; prescutum orange-yellow with three light brown stripes, sometimes not clear; wings pale brown with a dark brown stigma; abdomen orange with segments six and seven black; hypopygium orange, tergite nine divided by a V-shaped notch, produced into a pair of ear-like processes, sometimes terminated into spinous point, ventral-lateral portions of tergite nine projected into two lobes, densely covered with black setae.

#### Redescription.

Male. Length: *Body*: 14.0–18.0 mm (not including antenna, n = 5); *Wing*: 18.0–20.0 mm (n = 5); *Antenna*: 18.0–20.0 mm (n = 5).

**Figures 1–11.**
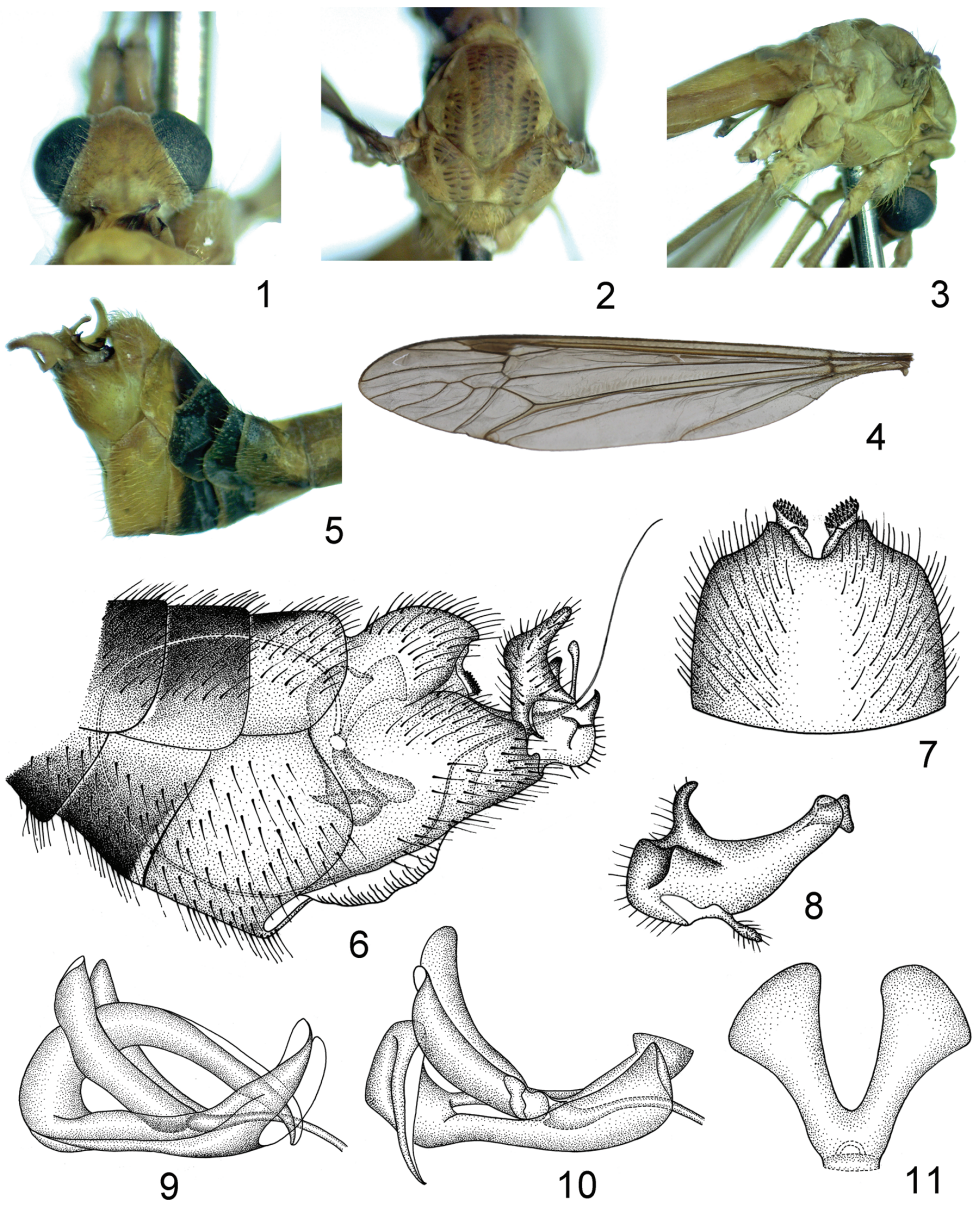
Tipula (Sivatipula) parvauricula **1** head, dorsal view **2** thorax, dorsal view **3** thorax, right lateral view **4** wing **5** hypopygium, right lateral view **6** perspective hypopygium, left lateral view **7** ninth tergite, dorsal view **8** inner gonostylus, left lateral view **9** and **10** semen pump, left lateral view **11** compressor apodeme, dorsal view.

*Head* orange. Rostrum orange with distinct orange nasus. Marking of vertex absent (Fig. 1). Eyes black (Fig. 1). Antenna: 12-segmented, distinctly longer than body; scape orange, expanded apically; pedicel orange, short; flagellomeres dark brown, each flagellomere cylindrical and subequal in length, with abundant black verticils, longest one longer than one third of flagellomeres length. Palpi light brown.

*Thorax* with pronotum entirely orange-yellow (Figs 2–3). Prescutum with three light brown stripes, sometimes not clear (Fig. 2). Scutum orange-yellow with two light brown stripes (Fig. 2). Scutellum and postnotum orange-yellow (Fig. 2). Pleura entirely bright yellow (Fig. 3). Legs slender, coxae, trochanters and femora orange-yellow, tibiae orange-yellow at basal half, changing to brown at apical half, tarsi brownish black. Halteres with stem yellow, knob darker. Wings pale brown, cell sc darker than ground color, stigma dark brown (Fig. 4). Sc relatively short, subequal to R_3_ in length, petiole of cell m1 as long as or slightly longer than m-m, approximately one-half length of discal cell (Fig. 4).

*Abdomen* orange with segments six and seven black. Hypopygium orange, broad, compressed (Figs 5–6). Tergite nine separated by a V-shaped notch in ventral view; gradually narrowed to apex, produced into a pair of ear-like processes, sometimes terminated into spinous point in lateral view; ventral-lateral portions of tergite nine projected into two lobes, densely covered with blackened setae (Figs 5–7). Sternite nine broader than tergite nine, median region of sternite nine protruded to a membranous extension (Fig. 6). Outer gonostylus flattened, widened medially, with a slender rod on ventral-lateral margin, directed dorsally (Fig. 6). Inner gonostylus broad basally, gradually narrowed to apex, rounded with a process truncated apically, ventral margin with a horn-shaped projection, dorsal margin with a finger-like process, basal region of inner gonostylus with a pyramidal process (Fig. 8).

*Semen pump* with compressor apodeme V-shaped, the arms expanded at apex (Fig. 11). Posterior immovable apodeme with only one arm, distinctly longer than compressor apodeme, gradually narrowed to apex and curved cephalad in lateral view (Fig. 9), sometimes abruptly bent to ventral margin (Fig. 10), the arm deeply grooved in dorsal view. Anterior immovable apodeme flattened, shorter than compressor apodeme, gradually narrowed to apex in lateral view (Figs 9–10). Aedeagus elongated, tubular, at least ten times longer than semen pump (Fig. 6).

#### Material examined.

**CHINA**: Guangxi Zhuang Autonomous Region: 2 males, Dalongping, Cenwanglaoshan Mountain, 24°31'N, 106°17'E, 1300 m, 11 May 2015, Guo-Xi Xue leg.; Guizhou Province: 3 males, Leigongshan Mountain, 26°21'N, 108°13'E, 4 Jun. 2015, Guo-Xi Xue leg.

#### Distribution.

China (Fujian, NW. Guangxi, SE. Guizhou).

#### Remarks.

In the original description of Tipula (Sivatipula) parvauricula (Alexander 1941), the prescutum is unmarked and the tergite nine is terminated into a pair of spinous points in lateral view. After observing five specimens, we noticed that the prescutum generally has three light brown stripes and the spinous point on tergite nine is not always present.

Three types of semen pumps were defined by Frommer (1963) based on morphological studies of the reproductive system of North American crane flies. Type III is the most common type characterized by the strongly bowed intromittent organ and by posterior immovable apodeme generally with two arms (Frommer 1963). According to the overall morphology, the semen pump of Tipula (Sivatipula) parvauricula should belong to Type III. However, its posterior immovable apodeme has only one arm, which differs from the results in previous works in Chinese species (*Ctenophora fumosa* Men, 2014 in Men and Huang 2014; Tipula (Vestiplex) coxitalis Alexander, 1935, Tipula (Pterelachisus) biaciculifera Alexander, 1937and Tipula (Emodotipula) yaoluopingensis Men, 2015 in Men 2015; Tipula (Yamatotipula) nova Walker, 1848 in Men et al. 2015a; *Nephrotoma liankangensis* Men, Xue & Yang and *Nephrotoma pseudoliankangensis* Men, Xue & Yang in Men et al. 2015b). This may represent a special form of type III.

### Tipula (Sivatipula) biprocessa sp. n.

Taxon classificationAnimaliaDipteraTipulidae

http://zoobank.org/0121BA77-9947-486A-91B8-15EAABEEFAB8

Figs 12–23

#### Diagnosis.

Generally straw-yellow; prescutum straw-yellow with three light brown stripes; wings pale brown with a dark brown stigma; abdomen bright yellow on basal three segments, gradually changed to light brown on apical ones, segments six and seven suffused with black; hypopygium straw-yellow, tergite nine rounded at posterior margin and equipped with two finger-like processes, ventral-lateral portions of tergite nine projected into two lobes, densely covered with black setae.

#### Description.

Male. Length: *Body*: 14.0–15.0 mm (not including antenna, n = 2); *Wing*: 19.0–20.0 mm (n = 2); *Antenna*: 18.0–19.0 mm (n = 2).

**Figures 12–23.**
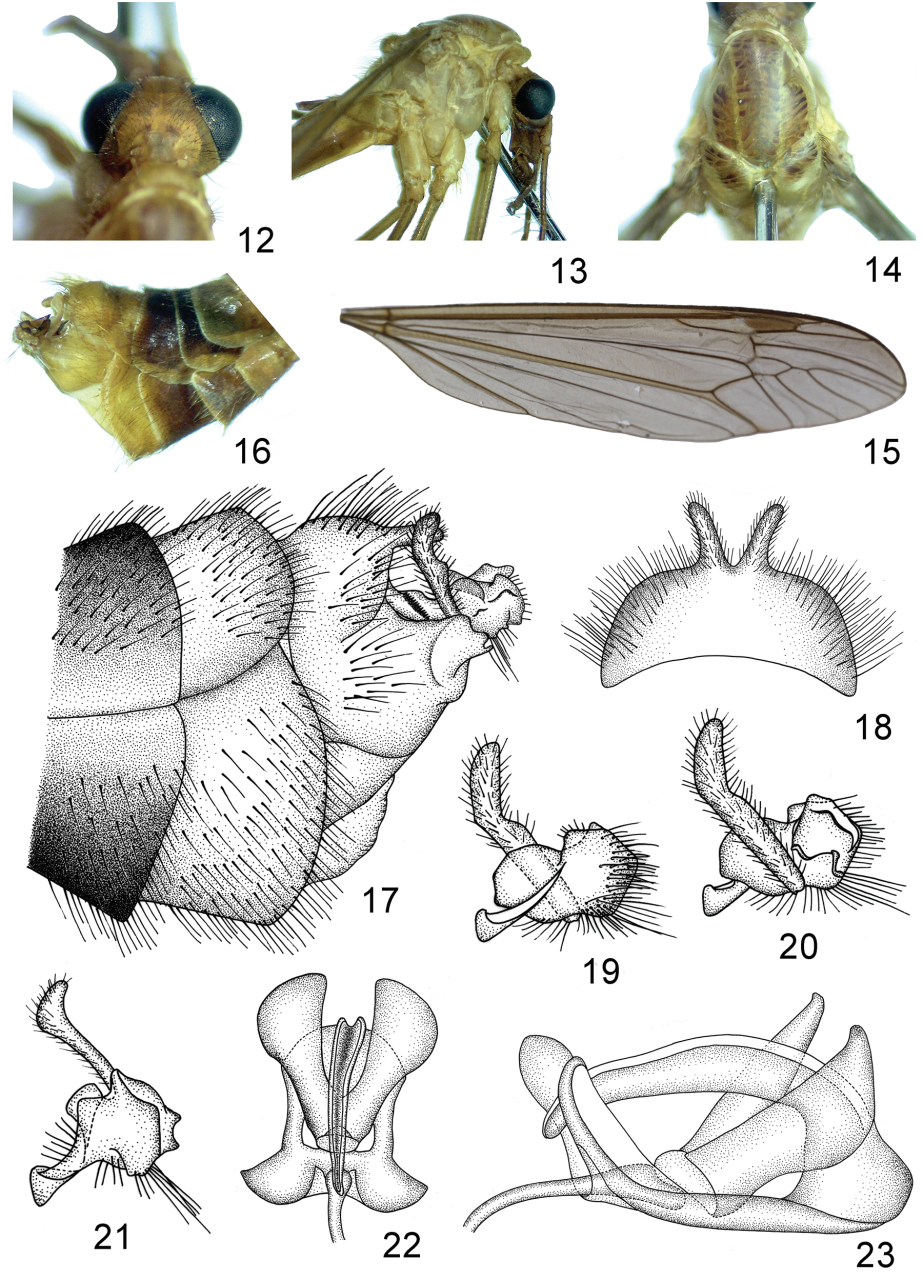
Tipula (Sivatipula) biprocessa sp. n. **12** head, dorsal view **13** thorax, right lateral view **14** thorax, dorsal view **15** wing **16** hypopygium, right lateral view **17** hypopygium, left lateral view **18** ninth tergite, dorsal view **19** inner gonostylus and outer gonostylus, left view **20** inner gonostylus and outer gonostylus, right view **21** inner gonostylus and outer gonostylus, dorsal view **22** semen pump, dorsal view **23** semen pump, right lateral view.

*Head* straw-yellow (Figs 12–13). Rostrum light brown with a light brown nasus. Vertex without marking (Fig. 12). Eyes black (Fig. 12). Antenna: 12-segmented, distinctly longer than body; scape light yellow, expanded apically; pedicel light yellow, short; flagellomeres light brown, each flagellomere cylindrical, subequal in length, with abundant black verticils. Palpi light brown.

*Thorax* with pronotum entirely orange-yellow (Figs 13–14). Prescutum straw-yellow with three light brown stripes (Fig. 14). Scutum orange-yellow with two light brown stripes (Fig. 14). Scutellum and postnotum orange-yellow (Fig. 14). Pleura entirely bright yellow (Fig. 13). Legs slender, coxae and trochanters straw-yellow, femora straw-yellow with light brown tip, tibiae and tarsi light brown. Halteres with stem yellow, knob darker. Wings pale brown, cell sc darker than ground color, stigma dark brown (Fig. 15). Sc relatively short, subequal to R_3_ in length, petiole of cell m1 slightly longer than m-m, approximately one-half length of discal cell (Fig. 15).

*Abdomen* bright yellow on basal three segments, gradually changed to light brown on apical ones, segments six and seven suffused with black (Fig. 16). Hypopygium straw-yellow (Fig. 16). Hypopygium broad, compressed (Figs 16–17). Tergite nine rounded at posterior margin with two finger-like processes, lateral sides of tergite nine with numerous long hairy setae, longest one longer than finger-like process (Fig. 18). Sternite nine broader than tergite nine, median region of sternite nine protruded to a membranous extension (Fig. 17). Outer gonostylus narrow, flattened, apical two-fifths curved caudad (Figs 19–21). Inner gonostylus flattened, narrowed medially, a slender lobe generated from the median region of inner gonostylus (Figs 19–21).

*Semen pump* with compressor apodeme V-shaped, the arms expanded at apex, distinctly broader than that of Tipula (Sivatipula) parvauricula (Fig. 22). Posterior immovable apodeme with one arm, distinctly longer than compressor apodeme, gradually narrowed to apex and curved cephalad in lateral view, the arm deeply grooved in dorsal view, basal region distinctly wider than that of Tipula (Sivatipula) parvauricula (Figs 22–23). Anterior immovable apodeme flattened, gradually narrowed to apex in lateral view (Fig. 22). Aedeagus elongated, tubular, at least ten times longer than semen pump.

#### Material examined.

**Holotype** male. **CHINA**: Guangxi Zhuang Autonomous Region, Dalongping, Cenwanglaoshan Mountain, 24°31'N, 106°17'E, 1300 m, 7 May 2015, Guo-Xi Xue leg. **Paratype.** 1 male, same data as holotype.

#### Remarks.

We compared the new species with all known species based on published descriptions and illustrations, and found that it is mostly similar to Tipula (Sivatipula) parvauricula by the color of body, the structures of antenna and hypopygium. It can be easily distinguished from the latter by the shape of tergite nine which is rounded at posterior margin with two distinct finger-like processes. The latter species has its tergite nine separated by a V-shaped notch with two short truncated processes and produced into a pair of ear-like processed ventrally. There is also a noticeable difference in the shape of the outer gonostylus which is simple and narrowed in the new species, but flattened and widened medially with a slender rod on ventral-lateral margin in that of Tipula (Sivatipula) parvauricula. Distinct interspecific difference is also found in the shape of inner gonostylus as Figures 8, 19 and 20.

#### Etymology.

The specific epithet is a noun derived from the Latin ‘*processa*’ with Latin prefix ‘*bi*’, referring to the presence of two finger-shaped processes at posterior margin of tergite nine.

#### Distribution.

China (NW. Guangxi).

### Key to species of subgenus Tipula (Sivatipula)

**Table:** 

1	Hind margin of ninth tergite rounded apically with a pair of finger-like processes (see Figs 16–18)	**Tipula (Sivatipula) biprocessa sp. n.** (China: Guangxi, Cenwanglaoshan Mountain. Fig. 24)
–	Hind margin of ninth tergite without such process	**2**
2	Outer gonostylus with an appressed pubescence on outer surface of apical arm (see Alexander 1928: 459, Pl. 2, fig. 5)	**Tipula (Sivatipula) lackschewitziana Alexander, 1928** (China: Taiwan, Noko Moutain. Fig. 24)
–	Outer gonostylus without such pubescence	**3**
3	Ninth tergite laterally bearing two spinous projections, lower to them submedially with two short spiculose projections (see Joseph, 1974: 277, figs 137–142)	**Tipula (Sivatipula) filicornis Brunetti, 1918** (India: West Bengal, Dajeeling. Fig. 24)
–	Ninth tergite not as above	**4**
4	Antenna slightly shorter than body	**5**
–	Antenna equal to or longer than body	**6**
5	Outer gonostylus club-shaped (see Alexander, 1953: 348, fig. 12d)	**Tipula (Sivatipula) pullimargo Alexander, 1951** (Myanmar: Adung Valley. Fig. 24)
–	Outer gonostylus long-attenuate (see Alexander 1940: 110, fig. 11)	**Tipula (Sivatipula) suensoniana Alexander, 1940** (China: Zhejiang, Tianmushan Mountain. Fig. 24)
6	Ninth tergite produced into a rounded apex, with numerous black setae (see Alexander 1971: 81, fig. 6)	**Tipula (Sivatipula) alhena Alexander, 1953** (Thailand: Chiengmai, Dio Suthep. Fig. 24)
–	Ninth tergite not produced into a rounded apex	**7**
7	Ninth tergite with powerful lateral arms that are tipped with abundant blackened pegs (see Alexnader 1964: 105, Pl. 4, fig. 40)	**Tipula (Sivatipula) bhishma Alexander, 1964** (India: Assam, Manipur. Fig. 24)
–	Ninth tergite without such pegs	**8**
8	Median lobe of ninth sternite bearing terminal brush-like setae (see Alexander 1927: 182, fig. 3)	**Tipula (Sivatipula) mitocera Alexander, 1927** (India: West Bengal, Dajeeling. Fig. 24)
–	Median lobe of ninth sternite without such brush-like setae (see Alexander 1941: 401, Pl. 4, fig. 44; Fig. 6)	**Tipula (Sivatipula) parvauricula Alexander, 1941** (China: Fujian, Wuyishan Mountain; Guangxi, Cenwanglaoshan Mountain; Guizhou, Leigongshan Mountain. Fig. 24)

**Figure 24.**
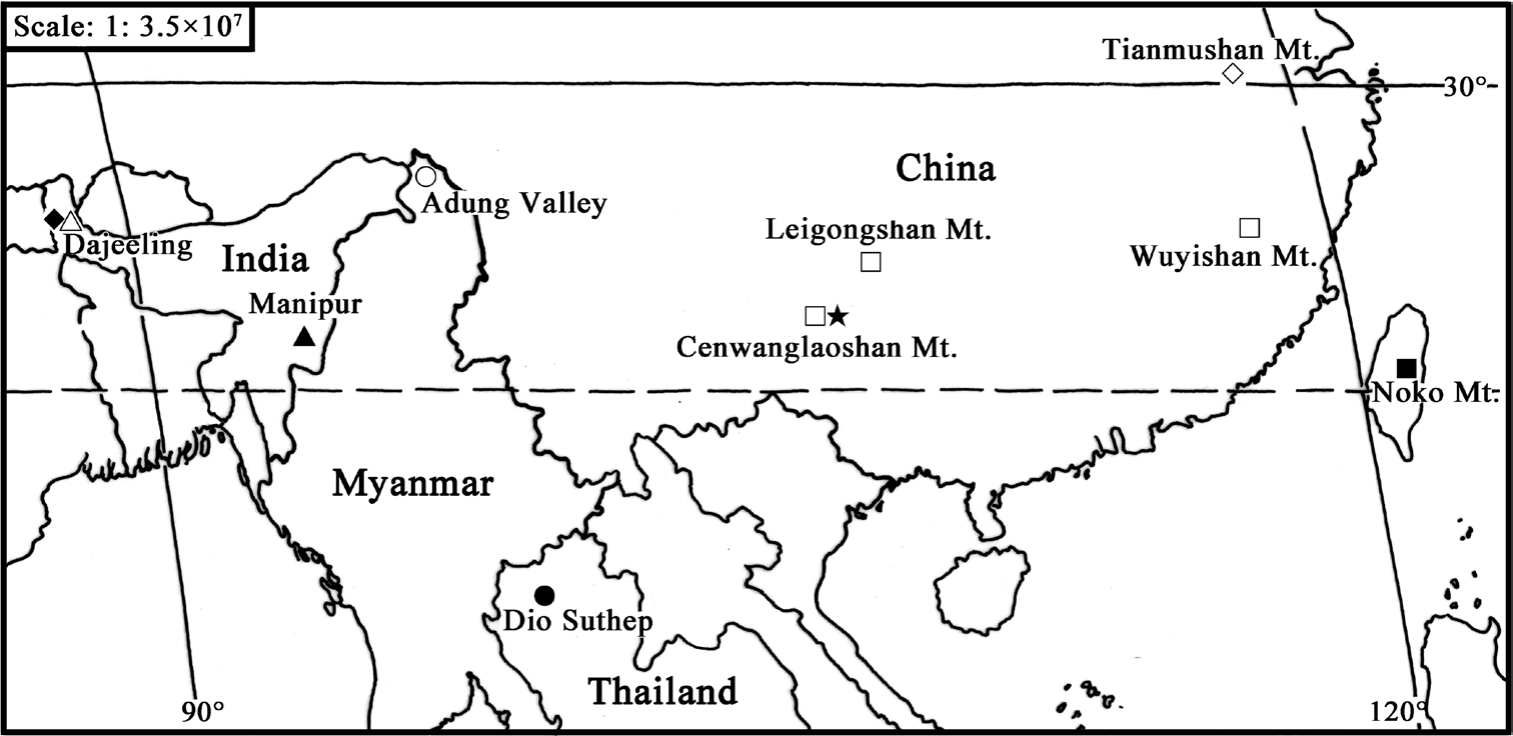
Geographic distribution of Tipula (Sivatipula) species: Tipula (Sivatipula) alhena (●), Tipula (Sivatipula) bhishma (▲), Tipula (Sivatipula) filicornis (◆), Tipula (Sivatipula) lackschewitziana (■), Tipula (Sivatipula) mitocera (△), Tipula (Sivatipula) pullimargo (○), Tipula (Sivatipula) suensoniana (◇), Tipula (Sivatipula) parvauricula (□), Tipula (Sivatipula) biprocessa sp. n. (★).

## Supplementary Material

XML Treatment for Tipula (Sivatipula) parvauricula

XML Treatment for Tipula (Sivatipula) biprocessa
